# The co-design, implementation and evaluation of a serious board game ‘PlayDecide patient safety’ to educate junior doctors about patient safety and the importance of reporting safety concerns

**DOI:** 10.1186/s12909-019-1655-2

**Published:** 2019-06-25

**Authors:** Marie Ward, Éidín Ní Shé, Aoife De Brún, Christian Korpos, Moayed Hamza, Elaine Burke, Ann Duffy, Karen Egan, Una Geary, Catherine Holland, Julie O’Grady, Karen Robinson, Alan Smith, Alan Watson, Eilish McAuliffe

**Affiliations:** 1 School of Nursing, Midwifery and Health Systems, College of Health Sciences, University College Dublin, Belfield, Dublin 4, Ireland; 2 St. James’s Hospital, Dublin 8, Ireland; 3 Clinical Risk, State Claims Agency, Grand Canal Street, Dublin 2, Ireland; 4 Patient Representative, Patient and Public Involvement in Healthcare at Health Service, Dublin 2, Ireland; 5 Mater Misericordiae University Hospital, Eccles Street, Dublin 7, Ireland; 6 St. Vincent’s University Hospital, Dublin 4, Ireland; 7 Health Sciences Centre, School of Nursing, Midwifery and Health Systems, College of Health Sciences, University College Dublin, Belfield, Dublin 4, Ireland

**Keywords:** Medical education, Junior doctors, Embedded learning, Serious game, Patient safety, Safety culture

## Abstract

**Background:**

We believe junior doctors are in a unique position in relation to reporting of incidents and safety culture. They are still in training and are also ‘fresh eyes’ on the system providing valuable insights into what they perceive as safe and unsafe behaviour. The aim of this study was to co-design and implement an embedded learning intervention – a serious board game – to educate junior doctors about patient safety and the importance of reporting safety concerns, while at the same time shaping a culture of responsiveness from senior medical staff.

**Methods:**

A serious game based on the PlayDecide framework was co-designed and implemented in two large urban acute teaching hospitals. To evaluate the educational value of the game voting on the position statements was recorded at the end of each game by a facilitator who also took notes after the game of key themes that emerged from the discussion. A sample of players were invited on a voluntary basis to take part in semi-structured interviews after playing the game using Flanagan’s Critical Incident Technique. A paper-based questionnaire on ‘Safety Concerns’ was developed and administered to assess pre-and post-playing the game reporting behaviour. Dissemination workshops were held with senior clinicians to promote more inclusive leadership behaviours and responsiveness to junior doctors raising of safety concerns from senior clinicians.

**Results:**

The game proved to be a valuable patient safety educational tool and proved effective in encouraging deep discussion on patient safety. There was a significant change in the reporting behaviour of junior doctors in one of the hospitals following the intervention.

**Conclusion:**

In healthcare, limited exposure to patient safety training and narrow understanding of safety compromise patients lives. The existing healthcare system needs to value the role that junior doctors and others could play in shaping a positive safety culture where reporting of all safety concerns is encouraged. Greater efforts need to be made at hospital level to develop a more pro-active safe and just culture that supports and encourages junior doctors and ultimately all doctors to understand and speak up about safety concerns.

**Electronic supplementary material:**

The online version of this article (10.1186/s12909-019-1655-2) contains supplementary material, which is available to authorized users.

## Background

Healthcare organisations have a responsibility for ensuring safe care and that governance of workplace settings creates a culture that supports good professional practice. Several authors have identified barriers to organisational learning [e.g]. [1, 2] and in particular, there is evidence of a blame culture in healthcare [3] and a number of high profile reports have identified a fear of speaking up about safety [4, 5].

Junior doctors (defined here as those in their intern year) and Senior House Officers (SHOs), in the acute hospital setting provide day-to-day care for patients while under the supervision of Registrars and Consultants. Historically junior doctors were perceived as being a ‘high risk’ group bringing little experience, often provided with limited and inadequate supervision and experiencing high stress levels [6]. More recently authors have outlined the potential of involving junior doctors in improving patient care [7, 8] and in preventing, reporting and learning from near misses and adverse events [9]. We contend that junior doctors are in a unique position in relation to reporting of incidents and safety culture. They are still in training and are also ‘fresh eyes’ on the system providing valuable insights into what they perceive as safe and unsafe behaviour. Thus, it is an opportune time to educate and inform junior doctors about safety from a systems perspective and their role in preventing harm through the reporting of safety concerns [10].

Hooper et al. [11] note however that when educated on the importance of reporting, junior doctors are responsive, but this does not translate into increased reporting behaviour as junior doctors did not perceive their senior medical colleagues to be supportive of reporting. Across most national healthcare systems there is a requirement for all healthcare staff to report near misses, incidents and accidents [e.g]. [12, 13]. Yet reporting rates in healthcare remain low and doctors have the lowest reporting rates at 2.6% compared to nursing/midwifery at 81.5% [14]. Mitchell et al.’s [15] review highlight multiple reasons for doctors’ reluctance to report including time constraints, medico legal fears, a lack of clarity about what to report, paucity of feedback regarding previously submitted reports, poor dissemination of the learning derived from incident reports and difficulties in using the data once disseminated.

Mitchell et al. [15] argue that it is this same disengagement that is preventing incident reporting from reaching its potential as a powerful patient safety tool. In the results of their interviews with international patient safety experts they noted “that by doctors abrogating their responsibility in reporting, the reports submitted were biased and missed important medically specific issues such as diagnostic errors” [p.95]. They highlight a number of issues to improve medical engagement in the reporting process including feeling safe in reporting, educating doctors about the importance of reporting and involving doctors in actions to improve safety based on outcomes of the reporting process.

The aim of this study was to increase awareness of safety and improve reporting behaviour amongst junior doctors. The research involved the co-design and implementation of an embedded learning intervention – a serious board game – to educate junior doctors (interns and SHOs) about patient safety and the importance of reporting safety concerns, while at the same time shaping a culture of responsiveness from senior medical staff [16].

## Methods

### Embedded learning and PlayDecide

Embedded learning is learning that is based on the premise that the more contextual learning is to the job or task, the more an individual is motivated to learn [17]. The embedded learning intervention developed and described here is a serious game. A serious game is a “game in which education (in its various forms) is the primary goal, rather than entertainment” [18]. The use of serious games in junior doctor education is not new [see reviews by e.g]. [19, 20]. Dankbaar et al. [21] compared traditional and serious computer-based game teaching methods to teach medical students about patient safety and found them to be equally efficient but the serious computer based game was more engaging for students.

One such serious game is PlayDecide, which is an open access discussion game, with a role-playing component, to talk in a simple and effective way about controversial issues [22–26]. The game consists of five different types of cards: story, white, information, challenge and issue cards. Story cards tell the game player a fictional narrative story of a character based on a real situation. A white card is a versatile blank card where a participant can write their own story or issue or information or opinion to present to the rest of the group. Information cards are factual cards that present up to date scientific information about the theme. Challenge cards are cards used by game players to stir up a conversation when the discussion stalls. Issue cards exhibit a range of perceptions, questions, and opinions on the overall theme of the game [26]. Research published utilising PlayDecide covers multiple topics [27–33]. We used the PlayDecide framework to develop a game to encourage junior doctors to raise and report issues of concern.

### Co-designing the PlayDecide patient safety (PPS) game content

Co-design in healthcare involves the equal partnership of individuals who work within the system (healthcare staff), individuals who have lived experience of using the system (patients and their families/carers) and the ‘designers’ of the new system (whether that be IT personnel in terms of electronic platforms to improve efficiency or researchers in terms of designing interventions to improve health systems) [34]. Co-design involves working together to design a new product, making full use of each other’s knowledge, resources and contributions, to achieve better outcomes or improved efficiency [35]. The benefits of adopting co-design principles in healthcare have been outlined by several authors [36–38].

A sub-group of people from the research project steering group was formed in April 2015 to co-design the PPS game content over a six-month period. This group consisted of eleven key stakeholders involved in the project with knowledge about patient safety from different perspectives. During the co-design process the lived experience of the participants in reporting, investigating, and managing incidents was interwoven with the patient representatives experience of healthcare incidents, State Claims Agency reports of incidents, and material presented from the national and international literature on patient safety [e.g. [10, 39], systems analysis of incidents [40, 41], medical professionalism [42] and the importance of speaking up [e.g.] [4, 5]. The co-design participants and process are outlined in Additional file 1: Appendix 1.

**Table 1:** List of position statements

1 All staff should report all concerns they have regarding patient safety, without fear of recrimination, in the knowledge that learning will happen and the system will be improved. Patient safety should be our top priority as healthcare professionals.	
2 All staff should report only serious concerns they have regarding patient safety without fear of recrimination, in the knowledge that learning will happen and the system will be improved in relation to serious concerns	
3 All concerns regarding patient safety should be reported, but only by senior members of staff. Reporting by more junior members of staff is less likely to be effective	
4 Staff cannot be expected to report safety concerns because they are too busy providing care. There is no value in reporting safety concerns if a patient wasn’t harmed or placed at risk. It is just a waste of people’s time and resources	

**Fig. 1:**
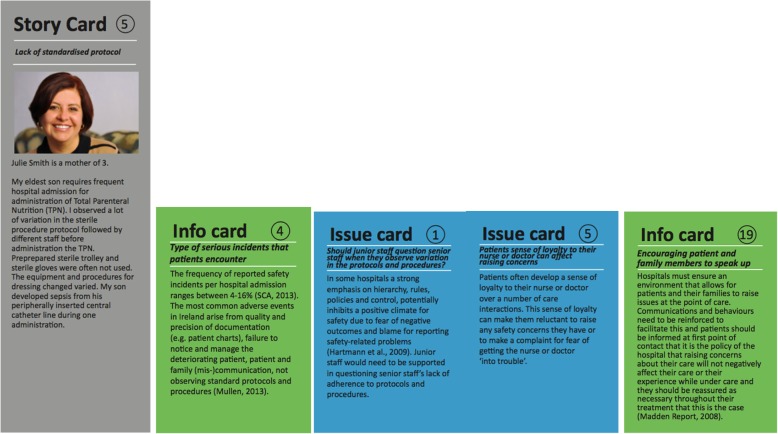
PlayDecide Patient Safety Game Sample Cards

Over three workshops, the content for 13 Story Cards, 22 Information Cards, 22 Issue Cards, 4 Position Statements and a Placemat was written. Story Cards, based on real incident cases and personal experiences of members of the co-design team, set the scene and provide the player with a first-hand account of a safety concern from different perspectives. Information Cards provide a fact related to patient safety. Issue Cards are based on key challenges and dilemmas in reporting safety concerns identified by the members of the co-design team. A sample of some of the cards developed is presented in Fig. 1.

Finally, position statements were also developed to represent a range of possible policy options from the ideal to the more practical. Voting on position statements by players is the final component of the PPS game playing and each group’s agreed position statement is recorded as an output of the game. Table 1 lists the position statements.

The game content was reviewed by a member of the project steering group who had only been able to attend the initial workshop and two external experts in the area of patient safety and Human Factors/Ergonomics to identify any gaps or areas for improvement. No additional changes were suggested by reviewers. The game was also tested for face validity and usability by the co-design team in a final workshop and minor changes were made to suggested timings and instructions and information for game participants. The full PPS game is an open source learning tool available to download for free by anyone interested in the game [43].

### Implementing the PPS game

#### Study sites, current reporting rates and participants

The PPS game has been specifically designed to help people discuss controversial issues in a safe manner. Each person is encouraged to read a sample of the story cards and then pick one that resonates with them. When all players have their story card picked then the players in turn summarise the story. A similar process happens for the information and issue cards. Each player picks two information and two issue cards and summarises them also for the rest of the players. The factual information cards and more nuanced issue cards that the players pick are usually related to the story card they have chosen thereby building up a larger narrative around the incident and what the causal and mitigating factors might have been. Allowing players to talk in the third person about safety incidents gives them a freedom to raise issues that they want to discuss.

The PPS game was played in two large urban acute academic teaching hospitals (Hospital A and Hospital B) in Ireland. In 2015, Hospital A introduced the electronic National Incident Management System (NIMS), which is accessible on the hospital internet system. Between 1/1/2015 and the 31/12/2015 there were 7973 incident reports submitted in Hospital A (Fig. 1). Nursing (including student nurses, staff nurses, Clinical Nurse Managers (CNM), Clinical Nurse Specialists (CNS), Advanced Nurse Practitioners (ANP), Assistant Director of Nursing (ADoN) and Healthcare Assistants (HCA)) reported 85.22% of all incidents. In comparison, medical staff reporting is extremely low with reports by doctors (Consultants and Non-Consultant Hospital Doctor (NCHDs)) accounting for only 3.74% of reports in 2015 and 3.09% in 2014.

**Fig. 2:**
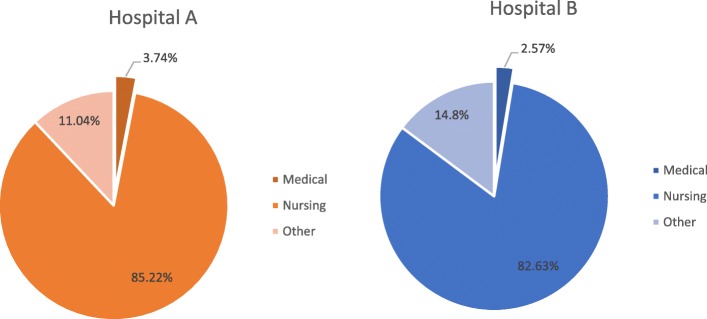
Hospital **a** and **b** incident reporting breakdown in 2015

Hospital B incident reporting system is paper based. Incidents are then entered onto the NIMS system. Between 01/01/2015 and 30/06/2016 3886 incidents were reported in Hospital B (Fig. 2). Nursing and Midwifery accounted for 82.63% of all reports submitted. In comparison to this medical staff only accounted for 2.57%. These figures, for both the nursing and medical categories, are comparable with the national reporting patterns for clinical adverse events reported above.

Members of the research team facilitated playing of the PPS game within each of the hospital sites. The study was carried out within the hospital educational and training centres where interns and SHOs were attending separate weekly lunchtime seminars. An initial meeting took place with interns and SHOs to explain the study within the two hospital sites. Participation was on a voluntary informed basis in line with the ethics process. The Intern Tutors acted as the gatekeepers for the interns in the hospital and information leaflets and consent forms were emailed to all interns seven days in advance of the first administration. Printed copies of the information leaflets and consent forms were given out on the first day of administration and at subsequent sessions for any interns who had missed the previous session/s. The NCHD Lead acted in a similar manner for the SHOs and a similar protocol was followed.

### Evaluating the educational impact of the game

To evaluate the educational value of the game voting on the position statements was recorded at the end of each game by the facilitator. The researchers also took notes after the game of key themes that emerged from the discussion. A sample of players were invited on a voluntary basis to take part in semi-structured interviews after playing the game using Flanagan’s Critical Incident Technique (CIT) [44]. CIT has been described as a systematic, inductive and flexible qualitative research method. It is a methodology for collecting and analysing data with the aim of providing solutions to practical problems [45]. The anonymous interviews explored participants understanding of incidents and captured suggestions from the interns and SHOs of what is required to shape a safety culture. The inductive approach of Thomas to analysing qualitative data was followed [46]. Pseudonyms are used to present the findings so as to protect the confidentiality of participants. NVivo 12 Pro was used to support the analysis. The interview schedule can be found in Additional file 2: Appendix 2.

### Evaluating the impact of the PPS game on reporting behaviour

Given that reporting rates for medical staff were so low in both hospitals, we developed a paper-based questionnaire on ‘Safety Concerns’ to establish a baseline of how many incidents junior doctors experienced or witnessed and how many they reported over the previous week. Four weeks of baseline data collection took place both before and after playing the PPS game. A similar process for administering the questionnaires was followed to playing the game where the Intern Tutor and NCHD Lead acted as gatekeepers.

The HSE 2014 definition of an incident was included in the questionnaire but the definition was extended to include ‘behaviour’ [47]. Many safety critical industries use behavioural based observations to explore safety levels and have shown a link between observed unsafe behaviours and injury levels [10].

The definition of incident used was thus “An event or circumstance *or behaviour* which could have, or did, lead to unintended and/or unnecessary harm. Incidents include adverse events which result in harm; near-misses which could have resulted in harm, but did not cause harm, either by chance or timely intervention; and staff or service user complaints which are associated with harm” [Adapted from 47p.5]. Examples given included: failure to maintain patient records, prescribing incorrect medication to patients, lack of communication between staff leading to lack of required care for patient, failure to adhere to standard protocols for sterile procedures, failure to detect sepsis, failure to notice patient deterioration, failure to provide information to a patient or their family. The Medical Council [48] model of the eight domains of professional practice was used to create a framework with which to attribute the potential contributory causes of the incidents. This model has been part of the intern training year since its development and is thus familiar to all junior doctors. These eight domains are: relating to patients; communication and interpersonal skills; collaboration and teamwork; management (including self-management); scholarship; professional conduct and ethics; clinical skills; relating to patients; patient safety and quality of patient care. Finally, there was a question on whether junior doctors, if they had witnessed a concern, had formally reported their concerns or informally discussed them with colleagues. The full questionnaire can be found in Additional file 3: Appendix 3.

In Hospital A the questionnaire was administered during the weekly educational sessions in which the intern doctors were required to participate. Administration commenced on 6th October 2015 and continued for four weeks. The PPS game was played with the interns in groups of 4–6 on 3rd November 2015. Following that the questionnaire was administered for a further four weeks. A similar process took place with the SHOs during their informal weekly teaching sessions however before and after the questionnaire administration was completed in 6 weeks. The PPS game was played with the SHOs on 30th November 2015.

In Hospital B data collection was undertaken with two cohorts of interns for a nine-week period. For the first cohort administration commenced on the 8th of April 2016 with the PPS game occurring on the 6th of May 2016. For the second cohort data collection commenced on 13th of September with the PPS game occurring on the 11th of October 2016. Efforts were made by the research team to engage SHOs in the project and efforts to enable this was supported by the NCHD lead. However no formal or informal teaching slots were available in Hospital B for SHOs therefore we were unable to play the PPS game with SHOs and data collection did not occur with this group.

#### Statistical analysis

Descriptive statistics will be used to explore incident reporting. Pearson’s chi-square tests (χ^2^) will be performed to assess: (i) if the intervention has an impact on whether an incident was witnessed; and (ii) to assess if the intervention has an impact on reporting behaviour. A Mann-Whitney test will also be conducted to determine any difference pre- and post-intervention in terms of the number of incidents or worrying behaviours interns and SHOs witness. IBM SPSS 24 will be used to support the analysis.

### Evaluating the impact of the PPS game on leadership inclusiveness and psychological safety

A brief survey of interns and SHOs was carried out to measure leader inclusiveness and psychological safety [49]. Again four weeks of pre- and post-playing the PPS game data collection was planned.

#### Leader inclusiveness

Leader inclusiveness refers to the behaviours and attitudes of the clinicians-in-charge. A three-item scale used by Nembhard and Edmondson [49] assessed the extent to which leaders’ words and deeds indicate an invitation and appreciation for others as contributing members in a team endeavour. The first two items on their scale, ‘senior doctors encourage other members of the team to take initiative’ and ‘senior doctors ask for the input of team members that belong to other professional groups’, were adapted from Shortell et al [50] physician leadership scale. The third item, ‘senior doctors do not value the opinion of others equally’ (reverse scored), was developed for the Nembhard and Edmondson study [49]. The items were adapted for local use in this study in that ‘physician leadership’ and ‘physician’ were changed to ‘senior doctor’.

#### Psychological safety

To measure psychological safety five items from Edmondson’s [51] psychological safety scale were adapted to this context (‘members of this NICU’ was removed from the original and replaced with ‘members of this team’) and used to assess the extent to which respondents felt safe to speak up about issues or ideas regarding their work:

- Members of this team are able to bring up problems and tough issues.
- People in this unit are comfortable checking with each other if they have questions about the right way to do something.
- If you make a mistake on this team, it is often held against you. (reverse scored)
- It is difficult to ask other members of this team for help. (reverse scored)
- Working with members of this team, my unique skills and talents are valued and used.

#### Statistical analysis

Descriptive analyses will be performed on the data. Respondents’ agreement (1—strongly disagree, 7—strongly agree) on the scale items will be summed and averaged to provide an individual scale score for each respondent. A Cronbach alpha test to evaluate scale reliability will be performed. An independent samples t-test will be employed to assess whether there are significant differences in scale scores between Hospitals A and B.

### Dissemination workshops to improve clinical leadership responsiveness

Dissemination workshops with senior clinicians across both sites were held to introduce them to the PPS game, to disseminate the information arising from the pre-implementation surveys and the PPS game playing sessions. The purpose of the workshops with senior clinicians was to develop suggestions for encouraging learning from incidents and safety concerns that have been raised by junior doctors and to promote more inclusive leadership behaviours and responsiveness to junior doctors raising of safety concerns from senior clinicians.

## Results

The impact of playing the PPS game and the intervention with the senior clinicians were evaluated in terms of: junior doctors awareness about patient safety and the value of reporting safety concerns and incidents; junior doctors reporting behaviour; junior doctors’ responses to the leadership inclusiveness and psychological safety surveys; and responsiveness from senior clinicians.

### Playing the PPS game and the position statements chosen

Members of the research team (MW, ÉNS, CK, ADB, MH, EMA) facilitated playing of the PPS game within each of the hospital sites between October 2015 and October 2016 during Intern and SHO teaching slots. There was one facilitator and groups of 4–8 junior doctors per game. In Hospital A the PPS game was played with 57 junior doctors (*n* = 36 interns and *n* = 21 SHOs). In Hospital B, the PPS game was played with two cohorts of interns with a total of 44 interns taking part.

Descriptive statistics show that an overwhelming majority of junior doctors (98%) who played the PPS game voted for position statement 1 where patient safety is highlighted as the focus of all staff and the need for on-going learning to improve the system is emphasised (see Additional file 4: Appendix 4 Table 1). Percentages were calculated based on summing the responses to the different position statements. This is a clear indication of junior doctors’ desire to see reporting safety concerns and patient safety as a top priority.

#### Hospital a

Descriptive statistics show that in Hospital A Position 1 was most supported by 98.25% between the two groups followed by Position 2 at 81.48%. Position 3 and Position 4 were *not* acceptable. Again percentages were calculated based on summing the responses to the different position statements. (Additional file 4: Appendix 4 Table 1).

Interns from one of the PPS groups came up with an alternate position which states “*Senior members should help filter the concerns from junior staff and support serious concerns”* receiving the most support at 60%. SHOs from one of the PPS groups proposed the following position “*All staff should be comfortable/able to report without fear of recrimination, and all staff should make time to report*”. This statement received 100% support from that group.

#### Hospital B

In Hospital B when combined and the data aggregated Position 1 was the most supported overall at 97.8% (Additional file 4: Appendix 4 Table 2).

One intern group in Hospital B came up with an alternate position statement where *“All staff should report* *all reasonable concerns* *they have regarding patient safety, without fear of recrimination, in the knowledge that learning will happen, and the system will be improved. Patient safety should be out top priority as healthcare professionals.”* This position received 100% support from that group.

### PPS game discussions and CIT interviews

The thematic analysis of facilitator notes from the discussions that took place during the game is presented here along with findings from the thematic analysis of the Critical Incident Technique interviews that took place with interns and SHOs (*n* = 15).

#### Feedback on playing PPS game

After playing the game and in the follow-up CIT interviews with a sample of players, players noted that they found the game very beneficial to play and that it provided a great space to talk among their peers in an open way about safety. All the players reported learning about safety, and in particular a systems understanding of safety, and the importance of reporting safety concerns and incidents. Discussion emerged on key patient safety topics from the cards. For example, a source of frustration in both sites was the lack of consistency in Standard Operating Procedures (SOPs) and protocols across hospitals and even across wards within a hospital. Having to learn ‘the ways’ of a new hospital or ward was often a source of personal stress and conflict with colleagues for junior doctors. All of the junior doctors in this study were enthusiastic about the possible role they could play in improving safety but were also worried about time constraints on their work, which would suggest that patient safety is still seen as something extra to one’s work and not seen as ‘core business’.

The main themes arising during the PPS game sessions and the interviews are discussed next.

#### Perceptions about responsibility for reporting incidents

There was a widely-held view amongst junior doctors that completing an incident report form is the duty of nurses, not doctors. This view was reinforced by: (1) nurses offering to complete forms in situations where a junior doctor has witnessed an incident; (2) senior doctors not fulfilling their obligation to report incidents.

*“Nurses are definitely more inclined...They would encourage each other and help each other to maybe put in the incident report form and say ‘No that is definitively an incident you need to put that in. Listen you go put that in now and I will do your job’.”* Janet (Hospital A).

This was in contrast to a lack of visible reporting by their medical colleagues:

*“From the point of my internship I never had a Registrar or SHO or a Consultant that would tell me that they are going to report an incident or do one.”* Carli (Hospital B).

#### Lack of clarity / knowledge about how to report an incident / safety concern

Participants within both hospital sites indicated that they were unclear of the procedure to report an incident. Many felt that they could not approach senior clinical staff informally about their concerns. They reported not witnessing their medical superiors engaging in reporting safety concerns and therefore had no role models to emulate in this regard. Some could not recall having received any training on the process, whereas others recalled receiving a very brief training, which they described as a ‘token gesture’. Training on the incident report system is provided in both hospital sites during junior doctors’ induction week. The literature is clear however in highlighting that junior doctors can experience high levels of stress starting a new placement and are often overwhelmed with the amount of information they receive during induction [52]. This would suggest that induction week may not be the best period for training junior doctors on how to use the incident reporting system.

#### Lack of clarity about what happens to reports once they are submitted

Junior doctors reported being unclear about what happens to an incident report once it is raised. They were unaware of who receives and reviews the information, how it is utilised and whether it results in any action.

*“There would be a strong feeling that these forms we fill in just end up in a shredder.”* Angelo (Hospital B).

Many questioned the merits of reporting an incident when no feedback to the person who raised the concern would be provided (feedback is usually sent to the local area manager to be sent back to the reportee), an issue that has been focused upon extensively in the literature [53–55].

*“People want to see the reporting loop closed when you report and seeing an outcome on that...and I think sometimes there is not enough feedback on that in any environment I don’t think Hospital A is different to any other hospital or any large institution.”* Monika (Hospital A).

#### Fear of reporting / speaking up about senior colleagues’ behaviour

Some participants were concerned that they might be characterised as ‘trouble makers’ by senior colleagues and be exposed to adverse career outcomes if they raised concerns through incident reporting.

*“If there is an incident with someone more senior to you on your team that’s when you are least likely to, you know, to say anything...we cannot really critique people above us as a rule. It just doesn’t happen.”* Angelo (Hospital B).

All participants stressed that the reporting process should be about learning and not about apportioning blame.

#### Lack of time to report

Junior doctors also raised the issue of not having time to report and failing to treat it as a priority in their time-constrained day, particularly as they had not observed senior doctors reporting. Hospital B’s paper-based incident reporting system was also a significant source of frustration for junior doctors.

#### Feeling that they are powerless to change the system

Junior doctors felt that their action of raising one report would not change anything and wondered what the point of reporting was at all.

*“It’s just I suppose a bit depressing but I feel like we cannot change the system at all that there is no point of filling out one form.”* Mia (Hospital B).

#### Perceptions that the reporting process was being used against doctors

Junior doctors felt the reporting system was being used against them as a threat or weapon. Examples emerged of junior doctors being told by nurses that they would file an incident report in situations where the doctor was paged and did not, or could not, respond to the nurse’s call immediately. This caused concern amongst the doctors that they would have a ‘black mark’ on their record.

#### Suggestions to improve safety culture and learning from incidents

During the PPS game sessions junior doctors in both sites made a number of suggestions as to how safety culture could be enhanced. Participants stressed the need for changing the culture by providing support to junior doctors when they did raise concerns. They also stressed the importance of learning from mistakes and incidents. For example the junior doctors felt that frequent feedback sessions on regularly occurring incidents and the lessons to be learned from them via the protected teaching slots would be hugely beneficial.

### Impact on reporting behaviour in relation to safety concerns

The Safety Concerns questionnaire was administered to junior doctors in Hospital A and B.

#### Hospital a

In Hospital A, 46 of 52 interns took part (82.14%) and 31 of 52 SHOs took part (59.62%). A total of 225 Questionnaires on Safety Concerns were gathered over the course of data gathering in Hospital A (148 interns; 77 SHOs;) (152 Time 1 and 73 Time 2).

#### Hospital B

In Hospital B, 72 of 86 interns took part (83.72%). A total of 195 Questionnaires on Safety Concerns were gathered from interns over the course of data gathering in Hospital B (141 Time 1; 54 Time 2).

For details on reporting behaviour please see Table 2.

**Table 2:** Reporting behaviour of junior doctors

	Reporting Behaviour	To whom do junior doctors (interns and SHOs) report incidents **
Hospital A	Out of the 148 Questionnaires on Safety Concerns gathered in Hospital A from interns, 38 (25.7%) witnessed a safety concern or incident in the previous week with only 4 of these respondents reporting the incident (10.5%). SHOs – 32 SHOs (41.6%) witnessed a safety concern or incident in the previous week with 19 stating they reported the incident (59.4%)	In Hospital A, of the 4 interns that formally reported incidents 75% (*n* = 3) reported to nursing staff. One participant reported to an SHO and one used the formal reporting system. In contrast for SHOs, out of the 19 that formally reported incidents, 42.1% (*n* = 8) reported their concerns to a registrar, followed by 36.8% (*n* = 7) to consultants with nursing staff at 31.6% (*n* = 6).
Hospital B	In Hospital B out of 195 questionnaires gathered (all interns), 65 (33.3%) stated they witnessed a safety concern or incident in the previous week with 20 stating they reported the incident (30.8%)	Of the 20 that formally reported incidents 55% (*n* = 11) reported to a registrar; 25% (*n* = 5) to SHOs; 20% (*n* = 4) to consultants; and 10% (*n* = 2) to intern; 15% (*n* = 3) personally submitted incident form; 5% (*n* = 1) ensured that someone else filled the incident form.

Note: ** Participants may have formally reported one incident to more than one individual or using more than one reporting mechanism

#### Did reporting behaviour change after playing the PPS game?

##### Hospital a

Before testing the hypotheses for Hospital A, the relationship between the population samples (interns and SHOs) and the PPS game (pre/post) variables a Pearson’s chi-square was conducted to explore if there was any significant difference between groups (a significance level of 0.05 was used for all tests). The Pearson chi-square was non-significant χ^2^(1) =0.087, *p* = 0.768, suggesting there was no statistically significant difference between the intern and SHO groups and therefore they were pooled to assess the effectiveness of the PPS intervention. The best way to assess the effectiveness of the intervention is to look at Question 1, 2 and 4 of the Questionnaire on Safety Concerns (Additional file 3: Appendix 3) as they answer the questions:

- Did the game have a significant effect on increasing awareness about experiencing and/or witnessing an incident (Q1)
- Did the game have a significant effect on increasing the number of incidents experienced and/or witnessed by interns and SHOs (Q2)
- Did the game have a significant effect on increasing the rate of whether or not interns and SHOs reported incidents (Q4)

For question 1 (Q1) a Pearson’s chi-square was conducted to assess the relationship between the Q1 variable (did you witness an incident) and the PPS game (pre/post) variable. The result was not significant, suggesting no impact of the PPS game on the number who reported witnessing an incident, χ^2^(1) = 3.034, *p* = 0.082.

A total of 61.4% of participants (interns and SHOs) witnessed 1 incident per week according to question 2 (Q2) of the Questionnaire on Safety Concerns (Table 2). A Mann-Whitney found no significant difference pre and post intervention in terms of the number of incidents or worrying behaviours interns and SHOs witnessed (Q2) (*U* = 586.00; *p* = 0.908).

A Pearson’s chi-square was conducted to assess if the PPS game (pre/post) variable had a statistical effect on whether or not the respondents reported incidents (Q4). The Pearson chi-square was significant χ^2^(1) = 5.336, *p* = 0.021 with a small to medium effect size suggesting the PPS game had a positive impact on incident reporting. (Phi = 0.276).

##### Hospital B

Similar to the procedure described for Hospital A, the same analyses were conducted to explore the impact of the intervention among interns in Hospital B on awareness of events (Q1), number of events witnessed (Q2), and whether the intervention had an effect on increasing the rate of incident reporting (Q4).

To address Q1, a Pearson’s chi-square test was conducted to assess the relationship between the Q1 variable (did you witness an incident) and the PPS game (pre/post) variable. The results indicated no significant difference between pre and post intervention, χ^2^ (1) = 0.026, *p* = 0.872.

A Mann-Whitney test was conducted to explore the effect of the PPS game on the number of incidents experienced and/or witnessed (Q2). It can be concluded that the intervention did not have a statistically significant effect on the number of incidents or worrying behaviours interns witnessed (*U* = 291.5; *p* = 0.065).

A Pearson’s chi-square was conducted to assess if the PPS game had a statistical effect on whether or not the respondents reported incidents (Q4). In contrast to Hospital A, there was no significant difference between reporting of incidents between pre- and post-intervention, χ^2^(1) = 0.864, *p* = 0.353. Across time points, almost three-quarters of participants stated that they discussed concerns informally with others (*n* = 46; 74.2%).

It must be noted that in both hospitals due to staff turnover and the non-compulsory attendance at training sessions it was not possible to ensure that all participants in Time 2 surveys had played the PPS game. Therefore the above results should be interpreted with caution. Table 3 summarises these results.

**Table 3:** Reporting behaviour of junior doctors - Pre and post intervention reporting levels

	Hospital A Interns		Hospital A SHOs		Hospital B Interns	
Pre intervention	25 interns (25.3%) had witnessed an incident	0% formally reported it	16 SHOs (30.2%) witnessed an incident	9 of these (56.3%) formally reported it. 6 (66.7%) of them stated they received a satisfactory response while 11.1% stated that they did not.	47 interns (33.3%) had witnessed an incident	13 interns (27.7%) formally reported it
Post intervention	13 interns (26.5%) had witnessed an incident	4 (30.8%) reported it and 50% of those received a satisfactory response	16 SHOs witnessed an incident; representing an increased proportion of respondents (66.7%)	10 (62.5%) formally reported the incident, with 40% receiving a satisfactory response, 50% not receiving a satisfactory response	The percentage of the sample witnessing an incident remained constant at 33.3% (*n* = 18)	7 (38.9%) of those formally reported the incident

#### Perceptions about contributory factors to incidents

**Fig. 3:**
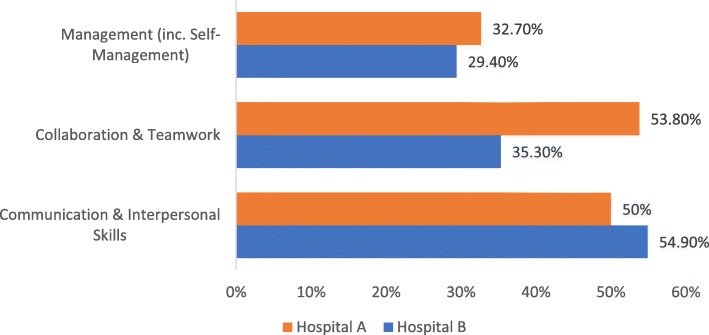
Top three contributory factors to incidents identified by junior doctors

As part of the Safety Concerns questionnaire, participants were asked to mark the aspect of the eight domains of Medical Professionalism they felt was a contributory factor in the safety concern or incident they had observed. In Hospital A the same top three contributory factors to incidents were selected by both interns and SHOs. Collaboration and teamwork was identified for SHOs (*n* = 17; 77.3%) while for interns 36.7% (*n* = 11) of the sample identified it as a contributory factor. The second most common factor for SHOs was communication and interpersonal skills (*n* = 12; 54.5%) which was the most common factor for interns at (*n* = 14; 46.7%). Thirdly, management (including self-management) was a factor for SHOs (n = 7;31.8%) and for interns (*n* = 10; 33.3%) (Fig. 3). In Hospital B the top three contributory factors to incidents were communication and interpersonal skills at 54.9% (*n* = 28) followed by collaboration and teamwork at 35.3% (*n* = 18) and management (including self-management) at 29.4% (*n* = 15).

### Leadership inclusiveness, psychological safety and responsiveness from senior clinicians

The original study plan was to administer the surveys, play the PPS game with the junior doctors and then hold the workshops with the senior clinicians before administering the post-implementation surveys. However in Hospital A only 8 out of 240 consultants attended the dissemination workshop and in Hospital B it was only possible to schedule the dissemination workshop after all the post-implementation surveys were administered. Thus we were not able to have any impact on responsiveness from the senior clinicians at all in Hospital A due to poor attendance and at the right time in Hospital B to be able to measure an impact in the post-implementation surveys. Therefore we did not administer the Time 2 leadership inclusiveness and psychological safety scales.

We had however measured leadership inclusiveness and psychological safety at baseline in both hospitals. Consistent with previous research [49] the leadership inclusiveness scale items were summed and averaged to provide a scale mean. In Hospital A (*n* = 74), the mean score observed was 5.39 (SD = 0.92) and the average total score in Hospital B (*n* = 71) was 5.2 (SD = 0.86) (out of a maximum total possible score of 7). There was no significant difference in mean scores between hospitals.

The Cronbach’s alpha reliability score was 0.43 which is lower than previous research and does raise a concern that we were reliably measuring leader inclusiveness. It would appear that question 3 ‘senior doctors do not value the opinion of others equally’, which was reverse scored, caused most tension in the responses. Please see Additional file 5: Appendix 5 Table 1.

### Psychological safety (5 item scale)

The five-item psychological safety scale was measured at baseline in both sites. Consistent with previous research [49] the scale items were summed and averaged to provide a scale mean. In Hospital A (*n* = 71), the mean score observed was 5.47 (SD = 0.79) and the average total score in Hospital B (*n* = 69) was 5.2 (SD = 0.98) (out of a maximum total possible score of 7). No significant differences between hospitals was observed and the scale demonstrated good reliability (Cronbach’s alpha = 0.78). The last question in this set ‘Working with members of this team, my unique skills and talents are valued and used’ showed the biggest variation. Please see Additional file 5: Appendix 5 Table 2.

From the consultants who did attend the dissemination workshop in Hospital A it was discerned that some did not know how the incident reporting systems worked and did not understand their potential role in creating a learning culture. Some points that emerged from the discussion include:

- The importance of closing the feedback loop for people who did raise incident reports, explaining what happened the report, what actions were taken, what was the hospital learning from it. There was agreement that it was essential to create a learning rather than a blaming culture and that senior consultants could play a role in this.
- Consultants could play a more proactive role in teaching junior doctors about safety, including; the use of anonymised incident reports for teaching purposes; giving their thoughts on incidents during ‘Grand Rounds’ or via the introduction of Safety Huddles; providing short summaries on incidents with key learning points; including patients’ perspectives and stories on incidents; undertake safety sessions during teaching studies-lunchtime seminars; introduce safety messages into clinical handovers.
- There was overall agreement that the induction week provided by the hospital was not the best time to train interns/SHOs on the incident reporting system due to the overload of information provided to them during that week. Suggestions were made that training should be provided more frequently and that the PPS game could be used for this purpose.

## Discussion

An embedded learning approach was taken to implement a serious game based on the PlayDecide framework to encourage junior doctors to speak up about and report safety concerns. The PlayDecide Patient Safety Game was co-designed by healthcare staff from the hospitals implementation sites, patient representatives and health systems researchers and proved to be an effective educational tool for encouraging deep discussion about patient safety and the importance of raising safety concerns. A significant strength of this study was the embedded learning approach taken and the authenticity of the PPS game material developed through the co-design process. The authenticity of the stories in particular helped to allow deep discussion to emerge very early in the game playing sessions.

From the voting on the position statements at the end of each game and the CIT interviews with a sample of participants we can see that the PPS game proved to be a valuable education tool about safety and in particular the importance of a systems approach to safety and proved to be effective in encouraging rich discussion and reflection on patient safety. A lack of understanding of Human Factors / Ergonomics (HF/E) and systems factors in healthcare has been noted and Durani et al. [56] note that junior doctors are significantly less likely to declare an understanding of the role of organisations in error management, suggesting that the organisational aspects of patient safety are less well embedded in patient safety exposure or training for junior doctors.

From the results of the Safety Concerns questionnaire we can conclude that junior doctors are witnessing behaviours and events that they find concerning. Some of them are reporting these to colleagues and fewer to their superiors. Junior doctors who do report are not using the formal incident reporting channels in the hospitals. The reasons stated for this include not feeling it is part of their role, not seeing their superiors report incidents, fear about reporting and feeling nothing will change as a result of reporting. These findings reflect those in the international literature. Kroll et al. [57] found that junior doctors commonly make and witness errors, some of which are serious, and that there is a prevailing norm of selective disclosure, which is likely to limit the systematic reporting of error. Lawton and Parker [58] note that the steep professional hierarchy in medicine inhibits the free reporting of experiences of error, rule violation or poor performance by junior doctors to their seniors because of the assumption that it would inhibit career development.

There was a significant change in the reporting behaviour of junior doctors in Hospital A following playing the PPS but not in Hospital B. This is consistent with our hypothesis that playing the game on its own was not meant to change behaviour but rather the game in combination with a change in leadership inclusiveness and subsequent psychological safety for the junior doctors would increase reporting. In Hospital A while we only managed to engage a small number of consultants in the dissemination workshop the results of the discussion demonstrated that consultants, rather than not supporting the incident reporting process, lacked awareness of the process and its importance in patient safety and their role in promoting the reporting of incidents, accidents and near misses among their junior colleagues. This was a very small sample of consultants however and we could not generalise based on these findings. Grimshaw et al. [59] make an interesting point in relation to doctors and the lack of translation of evidence into behavioural change arguing that doctors have not been trained or equipped with the skills to critically appraise the evidence base. The same argument could be made here in relation to a systems understanding of safety and improving and supporting culture change in relation to patient safety when senior doctors have not been trained in their role in this. A limitation of this study was that we were unable to engage with senior clinicians in Hospital B within the time between the pre and post measurement to encourage more responsiveness from them to both junior doctors reporting and modelling this behaviour themselves.

Also the significant difference in reporting behaviour in Hospital A does need to be interpreted with caution as there were far fewer surveys collected post-intervention and although a higher proportion of people who witnessed incidents reported them post-intervention, there was a lower response rate post-intervention (i.e., 9 out of 41 reported pre-intervention and 14 out of 29 reported post-intervention).

As we did not measure post-intervention psychological safety, we do not know if it improved for junior doctors following the game playing and the dissemination workshop. This is a limitation of our study and an area for future research. In a recent study Appelbaum et al. [60] found out in surveying resident physicians in the US that psychological safety was a predictor of intention to report adverse events and that perceived power distance and leader inclusiveness both influenced the intention to report adverse events through the concept of psychological safety. They did not measure actual reporting behaviour though. Senior clinicians are key to creating psychological safety for junior doctors to be able to speak openly about safety.

They do this in a number of ways, including subtle acts such as changing the language used in an organisation from threatening terms such as “errors” and “investigations” to more psychologically neutral terms such as “accidents” and “analysis” [61], by being more inclusive by means of words and deeds that appreciate others’ contributions [49], and by pardoning employees who disclose their unintentional mistakes [62] thereby creating a more just culture where learning from disclosure is encouraged and individual accountability for improvement is maintained [63]. When conditions are such that frontline staff feel free to speak up, they report more errors [64].

Studies have looked at building incident review and learning into Morbidity and Mortality (M&M) meetings [65]. Due to time constraints however M&M meetings are usually focused on in-hospital deaths only and not near misses, an understanding of which is essential for improving safety culture. In their study of teaching hospitals, Pierluissi and colleagues [66] found that errors were mentioned in at most 34% of M&M meetings (with some hospitals as low as 10%) and even more rarely were discussed in detail. When errors were discussed the result was “shaming and blaming” of an individual for the error [66]. M&M conferences, if carried out in this manner, reinforce not only the tendency to “shame and blame” but also individual accountability and individualised workarounds, both of which make it harder to sustain a safety culture. In contract the results demonstrate junior doctors identified ‘collaboration and teamwork’ and ‘communication and interpersonal skills’ as the top two contributory factors to incidents they witnessed. This highlights the need to work on and address precursors to incidents that arise from teamwork and communication at all opportunities rather than focus on individual mistakes and the adoption of evidence based HF/E team skills training programmes [e.g]. [67–69].

Participants in the PPS game were able to discuss safety issues in a safe manner as they were discussing them through a story, with information cards giving facts about patient safety and issue cards nuancing those facts with the day-to-day complexities of healthcare. If M&M meetings could be expanded to include such stories and more education on systems approaches to safety they could become a valuable source of learning for junior doctors.

Another limitation of this study was that by targeting the junior doctors via the weekly teaching sessions we did not capture all the doctors as the sessions were not compulsory, some had been on annual leave or unable to attend teaching due to clinical pressures. In Hospital B there was no organised SHO teaching slot, so instead we ran PPS game sessions with two intern cohorts. Our total sample is therefore smaller than originally planned and this has implications for the reliability and generalisability of our findings. Nonetheless, similar findings emerged across both sites and there was consistency in the qualitative data emerging from the discussions and the CIT interviews.

## Conclusion

In healthcare limited exposure to patient safety training and narrow understanding of safety compromise patients lives [70–72]. The existing healthcare system needs to value the role that junior doctors and others could play in shaping a positive safety culture where reporting of all safety concerns is encouraged. Training in a systems approach to patient safety, Human Factors/Ergonomics and the importance of reporting incidents, accidents and near misses needs to happen at all stages of medical education. Efforts need to be made at hospital level to develop a more pro-active safe and just culture that supports and encourages junior doctors and ultimately all doctors to understand and speak up about safety concerns.

## Additional files

Additional file 1: **Appendix 1.** Co-designing the PlayDecide Patient Safety Game. (DOCX 37 kb)

Additional file 2 **Appendix 2.** Imbuing Medical Professionalism in relation to Safety –Interview Questions. (DOCX 211 kb)

Additional file 3: **Appendix 3.** Questionnaire on Safety Concerns. (DOCX 298 kb)

Additional file 4: **Appendix 4.** Voting on Position Statements. **Table 1.** Hospital A Results: Percentages of how junior doctors voted for each position statement. **Table 2.** Hospital B Results: Percentages of how junior doctors voted for each position statement. (DOCX 14 kb)

Additional file 5: **Appendix 5. Table 1.** Item Statistics – Leader Inclusiveness. **Table 2.** Item Statistics – Psychological safety scale. (DOCX 14 kb)

## Data Availability

The PlayDecide Patient Safety Game is an open source embedded learning tool and can be found at: http://patientsafetydiscussions.ie/ The datasets used and/or analysed during the current study are available from the corresponding author on reasonable request.

## Abbreviations

ADoN: Assistant Director of Nursing
ANP: Advanced Nurse Practitioner
CIT: Critical Incident Technique
CNM: Clinical Nurse Manager
CNS: Clinical Nurse Specialist
HCA: Healthcare Assistant
HF/E: Human Factors/Ergonomics
HSE: Health Services Executive
M&M: Morbidity and Mortality
NCHD: Non-Consultant Hospital Doctor
NHS: National Health Service
NIMS: National Incident Management System
PPS: PlayDecide Patient Safety
SCA: State Claims Agency
SHO: Senior House Officer
SOP: Standard Operating Procedure
