# Supplementary material for: The co-design, implementation and evaluation of a serious board game ‘PlayDecide patient safety’ to educate junior doctors about patient safety and the importance of reporting safety concerns
Source: BMC Med Educ. 2019 Jun 25;19:232. doi: 10.1186/s12909-019-1655-2 (PMC6593521; doi:10.1186/s12909-019-1655-2)
Supplement: Supplementary file 1 — Appendix 1. Co-designing the PlayDecide Patient Safety Game. (DOCX 37 kb) [file 12909_2019_1655_MOESM1_ESM.docx]

Appendix 1: Co-designing the PlayDecide Patient Safety Game

**1. Background**

PlayDecide is a serious card based open access game, with a role-playing component to it where each person debates their view based on their selected story card. The game framework was created to allow players to discuss controversial issues in a safe environment. The game consists of five different types of cards: story, white, information, challenge and issue cards. Story cards tell the game player a fictional narrative story of a character based on a real situation. A white card is a versatile blank card where a participant can write their own story or issue or information or opinion to present to the rest of the group. Information cards are factual cards that present up to date scientific information about the theme. Challenge cards are cards used by game players to stir up a conversation when the discussion stalls. Issue cards exhibit a range of perceptions, questions, and opinions on the overall theme of the game [1].

The game consists of four to eight players, and each game has three phases that take a total of ~80 minutes to play. The first phase takes ~30 minutes where each player picks a story card in turn and summaries it to the group in turn. Similarly, each game player picks two information and issue cards in turn and summaries each. The second phase takes ~30 minutes of discussion among the players. If the discussion stalls, a player can use the challenge card to encourage further discussion. The third stage takes ~20 minutes where game players discuss four prewritten positions and vote on each of the positions in turn. The group can also devise their own fifth position if any one of the four does not encompass their group response.

**2. Study Participants**

A sub-group of people from the research project steering group was formed in April 2015 to co-design the PlayDecide Patient Safety Game content over a six-month period. This group consisted of eleven key stakeholders involved in the project with knowledge about patient safety from different perspectives. The group included three researchers (Professor of Health Systems, Senior Research Fellow in Health Systems & Human Factors/Ergonomics and a Research Assistant), a Patient Representative, a Clinical Risk Advisor from the State Claims Agency (SCA), a hospital Risk Manager, a hospital Quality and Safety Clinical Lead, a Non-Consultant Hospital Doctors (NCHD) lead, a hospital Patient Liaison Officer, the Nursing Quality, Audit and Research Co-ordinator, and the Medical Intern Tutor. Only some participants knew each other or the research team prior to the project commencing. Involvement in the game development and attendance at the workshops was on a voluntary basis. All members were familiar with the overall aim and objectives of the research project.

**3. Method: The Co-design Process**

Over six two-hour workshops the content for the PlayDecide Patient Safety game was co-designed by the 11 stakeholders. The PPS game was iteratively developed across five stages represented in Fig. 1.

The overall game framework, as outlined above, already exists and the game development manual proved an invaluable resource in customising the game for our purposes [2]. A sample game on Orphan Drugs was played in the first workshop to allow participants become familiar with the game structure [3].

Co-design in healthcare involves the equal partnership of individuals who work within the system (healthcare staff), individuals who have lived experience of using the system (patients and their families/carers) and the ‘designers’ of the new system (whether that be IT personnel in terms of electronic platforms to improve efficiency or researchers in terms of designing interventions to improve health systems) [4]. Co-design involves working together to design a new product, making full use of each other’s knowledge, resources and contributions, to achieve better outcomes or improved efficiency [5]. The benefits of adopting co-design principles in healthcare have been outlined by several authors [6-8].

During the co-design process the lived experience of the participants in reporting, investigating, and managing incidents was interwoven with the patient representatives experience of healthcare incidents and material presented from the national and international literature on patient safety [e.g. 9,10], systems analysis of incidents [11,12], medical professionalism [13] the importance of speaking up [e.g. 14,15]. Methods used within the co-design approach were for example using paper ‘stickies’ to allow each person to generate suggestions; facilitated discussions in pairs, small groups and the large group; presentation and discussion of sample material [4].

The purpose statement for the game was agreed upon as: “Creating a culture that empowers junior doctors to speak up about safety concerns”. A list of key messages to be communicated to junior doctors, via the game, were drawn up by the group. These included the following:

- Understanding the elements of a just and fair culture and the importance of speaking up;
- The importance of taking personal responsibility for safety;
- Understanding safety from a systems perspective and knowledge of the most common types of serious incidents that can happen and their frequency of occurrence;
- Learning about safety-I (learning from what goes wrong) and safety-II (learning from what goes wrong and what goes right/examples of excellence);
- Understanding perceptions of the personal risks to reporting including fear of retribution;
- The importance of reporting to enable organisational learning;
- Support to person who was involved in incident (second victim support);
- Reassuring junior doctors that mistakes are normal and need to be learned from

**4. Results: The PlayDecide Patient Safety (PPS) Game**

Over the course of the next three workshops a set 13 Story Cards, 22 Information Cards, 22 Issue Cards, 4 Position Statements and a Placemat were developed. Story Cards set the scene and provide the player with a first-hand account of a safety concern from different perspectives. Information Cards provide a fact related to patient safety. Issue Cards are based on key challenges and dilemmas in reporting safety concerns identified by the participants.

*Story Cards*

Suggested stories, which came from both personal and professional experiences including incidents reported to the SCA, were then discussed and individual’s recollection and sharing of further stories was sparked from the discussion. As trust built over the course of the workshops individuals shared more personal stories relating to the care they themselves or family members received in a hospital setting and stories from their own professional work in the hospitals. A set of 13 Story Cards was developed based on real cases from the SCA, from the hospitals involved and from the personal experiences of members of the group in relation to the care of either themselves or family members. Of the 13 Story Cards developed 12 of them relate to cases where patient safety was compromised. One card relates to the power of reporting to affect change.

*Information and Issue Cards*

The Information and Issue Cards play a pivotal role in the game as they serve to provide the players with additional perspectives that nuance the stories of patient safety raised in the Story Cards and the difficulties that are faced on a daily basis in relation to improving patient safety. The Vincent’s ‘seven levels of safety’ framework for the systems analysis of incidents was applied by the group to all 12 cases reflected in the Story Cards to help take a more systems perspective on each case. This along with the wealth of experience of the group members in relation to systems analysis, Human Factors/Ergonomics, incident investigation, risk management and organisational learning helped to develop the information and issue cards related to each story. A set of 22 Information and 22 Issue Cards relating to the Story Cards were developed. These reflect both national and international information and issues in relation to patient safety and to reflect the key messages that the group wanted the game to convey.

*Position Statements*

The Position Statements were developed at the fifth workshop. The game manual suggests having four statements, two on the extreme side and two middle positions. The co-design group found it difficult to word the position statements in this manner and finally agreed on the following statements, which reflect a continuum of views.

A final set of four Position Statements were developed:

1. All staff should report all concerns they have regarding patient safety, without fear of recrimination, in the knowledge that learning will happen and the system will be improved. Patient safety should be our top priority as healthcare professionals.
2. All staff should report only serious concerns they have regarding patient safety without fear of recrimination, in the knowledge that learning will happen and the system will be improved in relation to serious concerns.
3. All concerns regarding patient safety should be reported, but only by senior members of staff. Reporting by more junior members of staff is less likely to be effective.
4. Staff cannot be expected to report safety concerns because they are too busy providing care. There is no value in reporting safety concerns if a patient wasn’t harmed or placed at risk. It is just a waste of people’s time and resources.

**5. Piloting of the PPS game**

The FUND manual recommends that three people check the draft content of the game and look for possible biases in terms of which cards support which policy statements [2]. Thus the draft game was sent for review to three people: a member of the project steering group who had only been able to attend the initial workshop (she subsequently attended the last workshop), and to two external experts in the area of patient safety; an experienced incident investigator and a lecturer in Human Factors/Ergonomics in Healthcare. They were asked to review the existing content including the position statements and to highlight if they felt there were any areas missing that should have been included. The three reviewers all felt that the game as it stood was of good quality and did capture the complexity of issues surrounding patient safety.

At the final workshop the game was played by all attendees. All felt that the game framework worked well and that the content was good. Some changes were made to the timing of the game and it was also agreed to give extra instruction to those that already exist on the PlayDecide game. We were advised by the hospitals that the most time junior doctors would have to play in any one session was 1 hour so we adapted the times for this. Because of this time constraint it was agreed that an ideal number of players per game would be 6-8 people.

It was also agreed that each junior doctor who played needed to be informed of the following:

- The game is to educate and inform the participants. It is not to develop or inform policy.
- Their opinions are confidential. Nothing is recorded throughout the game except the final policy position of the group.
- If they have any serious concerns over safety, please report them.

**6. Discussion: Co-designing the PPS game, strengths and limitations**

The FUND PlayDecide manual proved an invaluable resource in supporting the technical development of the PlayDecide Patient Safety game by providing specific criteria for what type of information should be in the cards. The team also brought their personal and professional experience to develop the content of this particular game. This was both a particular strength and a challenge in terms of developing the game. All of us on the development group had both professional and personal experiences of the healthcare system and this emerged through the development of the Story Cards. At times there was a risk that discussions about people’s negative experiences of patient safety could spiral into negativity towards the healthcare system and a sense of helplessness to change or improve it.

The game structure however ensured we returned to our primary objective, which was to develop a game that would assist in “Creating a culture that empowers junior doctors to speak up about safety concerns”. For this purpose, we needed to keep focused on developing their sense of medical professionalism in relation to speaking up about safety but also through the game, and the Information and Issue Cards, to teach them about the importance of reporting safety concerns, the organisational learning that can happen and an understanding of their role in a larger preventative systems approach to safety. The diversity of the group members from junior doctors to risk managers to quality and safety specialists was crucial in this regard.

One weakness however was the poor attendance at the workshops of junior doctors. It was extremely difficult to have them released for the workshops, which were each two hours in duration. Also the hospital rotations took place in July, which was during the game development phase. The NCHD lead and the Intern Tutor did represent the junior doctors on the game development initially. They too however found it difficult to get released for the workshops. There were two non-practicing physicians on the group, which helped and in order to ensure the voice of the junior doctors in particular was heard we requested volunteers from within our Health Systems research team who had medical training. Through this process a colleague and recent medical graduate attended the last two workshops.

Acknowledgements:

We would like to acknowledge Dr. Eva Doherty DClinPsych, CClinPsych(AFPsI), CPsychol(AFBPsSI), Director of Human Factors in Patient Safety, Royal College of Surgeons in Ireland and Ms. Cora McCaughan, Assistant National Director, Healthcare Audit, Quality Assurance and Verification Division, HSE and formerly of the National Incident Management Team for reviewing the draft PlayDecide Patient Safety materials.

References

1. PlayDecide. About FUND and PlayDecide. N/A. <http://www.playdecide.eu/about>. [Accessed 14 September 2016].
2. PlayDecide. FUND Manual; 2010. Available at: <http://www.playdecide.eu/sites/default/files/instructions/Fund_Manual_4.2.pdf> (Last accessed 14 November 2016).
3. PlayDecide. Game on Orphan Drugs; 2010. Available at: <http://www.playdecide.eu/play/topics/orphan-drugs> (Last accessed 25 November 2016).
4. Ward ME, De Brún A, Conway C, Cunningham U, English A, Fitzsimons J, Furlong E, Kane Y, Kelly A, McDonnell S, McGinley S, Monaghan B, Myler A, O’Donovan R, O’Shea M, Shuhaiber A, McAuliffe E. Using co-design to develop a collective leadership intervention for healthcare teams to improve safety culture. Accepted: International Journal of Environmental Research and Public Health. Special Edition: Health Systems Research: New and Innovative Methods for Exploration and Implementation. 2018 Jun 5;15(6). pii: E1182. doi: 10.3390/ijerph15061182.
5. Bovaird T and Loeffler E. (2012), “From engagement to co-production: How users and communities contribute to public services” in Taco Brandsen and Victor Pestoff (eds.), New public governance, the third sector and co-production. London: Routledge.
6. Davies N, Mathew R, Wilcock J,  Manthorpe J, Sampson EL, Lamahewa K & Iliffe S. A co-design process developing heuristics for practitioners providing end of life care for people with dementia. BMC Palliative Care 201615:68 [doi:10.1186/s12904-016-0146-z](https://doi.org/10.1186/s12904-016-0146-z)
7. Blackwell (née Wright) R, Lowton K, Robert G, Grudzen C, Using Experience-based Co-design with older patients, their families and staff to improve palliative care experiences in the Emergency Department: A reflective critique on the process and outcomes. Int J Nurs Stud, 2017, 68, 83 – 94 doi: 10.1016/j.ijnurstu.2017.01.002.
8. Manikam L, Shah R, Reed K, Santini G and Lakhanpaul M. (2017), Using a co-production prioritization exercise involving South Asian children, young people and their families to identify health priorities requiring further research and public awareness. Health Expect, 20: 852–861. doi:10.1111/hex.12524
9. Kohn LT, Corrigan JM, Donaldson MS. To err is human: building a safer health system. Institute of Medicine (US) Committee on Quality of Health Care in America. Washington (DC): National Academies Press (US); 2000.
10. Vincent C, Burnett S, & Carthey J (2013). The measurement and monitoring of safety: drawing together academic evidence and practical experience to produce a framework for safety measurement and monitoring. The Health Foundation. Available at: <https://www.health.org.uk/sites/health/files/TheMeasurementAndMonitoringOfSafety_fullversion.pdf> (Last accessed 25 July 2018)
11. Health Service Executive. HSE Guidelines for Systems Analysis Investigation of Incidents and Complaints. Dublin: HSE; 2012. Available at: <http://www.hse.ie/eng/about/Who/qualityandpatientsafety/resourcesintelligence/Quality_and_Patient_Safety_Documents/QPSDGL5211.pdf> (Last accessed 22 November 2016).
12. Vincent C. Patient Safety. 2nd ed. Chichester: Wiley-Blackwell; 2010.
13. Medical Council. Talking about Good Professional Practice, views on what it means to be a good doctor. Dublin: Medical Council; 2014.

### Madden D. 2008 Building a Culture of Patient Safety – Report of the Commission on Patient Safety and Quality Assurance. Available at: <https://health.gov.ie/blog/publications/building-a-culture-of-patient-safety-report-of-the-commission-on-patient-safety-and-quality-assurance/> (Last accessed 25 July 2018)

1. Francis R. Report of the Mid Staffordshire NHS Foundation Trust Public Inquiry. London: The Stationery Office; 2013. Available at:

<https://www.gov.uk/government/publications/report-of-the-mid-staffordshire-nhs-foundation-trust-public-inquiry> (Last accessed 25 July 2018)
