# Supplementary material for: The co-design, implementation and evaluation of a serious board game ‘PlayDecide patient safety’ to educate junior doctors about patient safety and the importance of reporting safety concerns
Source: BMC Med Educ. 2019 Jun 25;19:232. doi: 10.1186/s12909-019-1655-2 (PMC6593521; doi:10.1186/s12909-019-1655-2)
Supplement: Supplementary file 2 — Appendix 2. Imbuing Medical Professionalism in relation to Safety –Interview Questions. (DOCX 211 kb) [file 12909_2019_1655_MOESM2_ESM.docx]

**Project Title:** Imbuing Medical Professionalism in relation to Safety –Interview Questions

**Theme 1: General Profile and Overall Experience**

- Can you please state the hospital you work in what department and your job title?
- Can you tell me how long you have been working in this hospital?
- How many other hospitals have you worked in?

**Theme 2: Interviewees understanding of an incident**

- In your opinion what are the characteristics of an incident?
- What is your understanding of the process of reporting an incident?

**Theme 3: Interviewees experience/observation of an incident**

Thinking back over the last six months did you observe or hear about any incident or a behavior in work that concerned you?

If answered **Yes:**

- Can you describe the sequence of events – what led up to the event? Who was present? Etc.
- How did you react? How did the people around you react? What was the impact on other team members?
- Was the incident reported and if so what reporting process was used and by whom? Was there an outcome? Was it implemented?
- Would you report the incident yourself? Why and why not?
- Based on your experience or knowledge of how this incident was handled, would it encourage you to report incidents in the future? Why or why not?

If Answered **No:**

- Thinking back over the last six months what were the reasons or factors that resulted in you not observing any incident?
- Based on your experience or knowledge if you had observed an incident that would have concerned you how would you have gone about reporting it? What process of reporting would you have used and who would you have reported it to? Would you report the incident yourself? Why or/why not?
- Does the current environment you work in encourages you to report any potential and informs you of the process?

**Theme 4: Suggestions for shaping a safety culture and a supportive environment**

- In your opinion are there any issues that act as barriers to you or your NCHD (non-consultant hospital doctors) colleagues reporting incidents?
- Are some health professionals more or less inclined to report incidents? Why?
- Are there any supports that the hospital offers that encourage NCHDs to report?

In your opinion considering your experience over the last six months what are the actions and activities you would propose to shape a safety culture and a supportive environment of reporting?
