# Supplementary material for: The co-design, implementation and evaluation of a serious board game ‘PlayDecide patient safety’ to educate junior doctors about patient safety and the importance of reporting safety concerns
Source: BMC Med Educ. 2019 Jun 25;19:232. doi: 10.1186/s12909-019-1655-2 (PMC6593521; doi:10.1186/s12909-019-1655-2)
Supplement: Supplementary file 3 — Appendix 3. Questionnaire on Safety Concerns. (DOCX 298 kb) [file 12909_2019_1655_MOESM3_ESM.docx]

**Questionnaire on Safety Concerns**

1. Over the course of the past week did you experience or witness any  incident/s or behaviour/s that would concern you in relation to patient care/safety?

Incident is an “An event or circumstance or behaviour which could have, or did lead to unintended and/or unnecessary harm. Incidents include adverse events which result in harm; near-misses which could have resulted in harm, but did not cause harm, either by chance or timely intervention; and staff or service user complaints which are associated with harm” (Adapted from HSE, 2014 p.5). Please see overleaf for examples.

YES☐ NO☐

If YES please proceed to question 2

1. If YES, how many incidents did you experience or witness? ______________________________________________

3. Do you think the contributory factors to these incidents were related to any aspects of professionalism outlined below, please place an X on the appropriate places in the drawing. Please see overleaf for further details on each category.


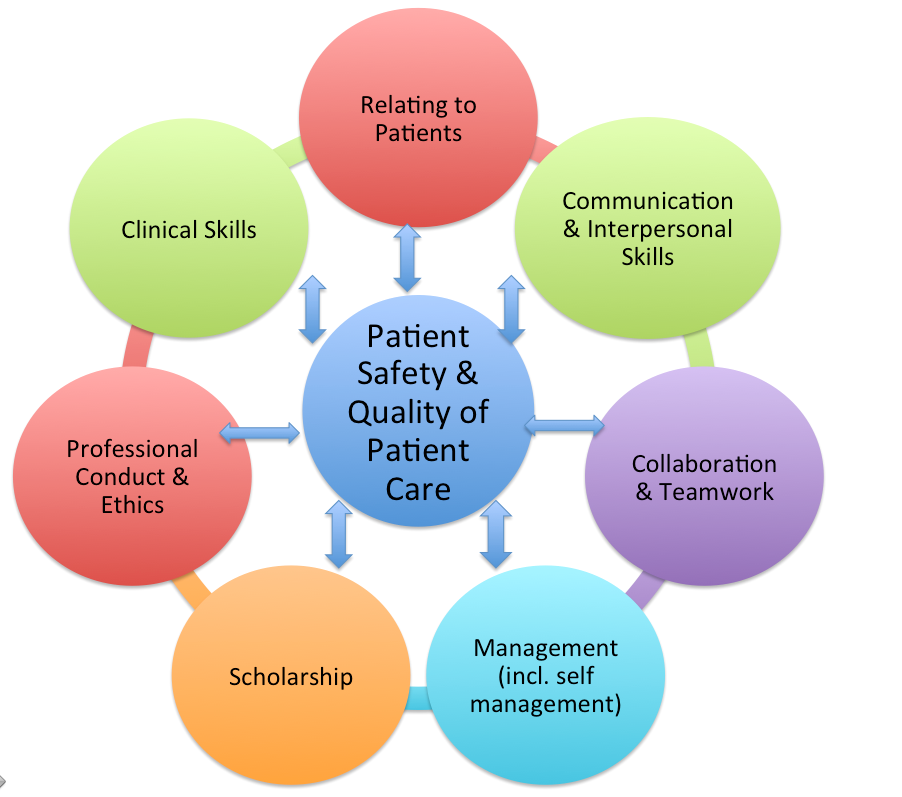


Please expand if you wish:

1. Did you formally report your concerns?

YES ☐ NO ☐ If NO please proceed to question 7

1. If YES, please indicate with whom / how you shared them (please tick all that apply):

| Intern ☐ | Intern tutor ☐ |
| --- | --- |
| SHO☐ | Lead NCHD ☐ |
| Reg ☐ | Health & Social Care Professional ☐ |
| Consultant ☐ | Personally submitted Risk/Adverse Incident Report form ☐ |
| Nursing staff member ☐ | Ensured someone filled out Risk / AIR form ☐ |
| Risk manager ☐ | Other, please specify: |

6. If YES, did you receive a satisfactory response? ___________________________________________________________

7. If NO, please specify why you did not formally report them? ____________________________________________

8. Did you informally discuss your concerns with anyone? YES ☐ NO ☐

9. If YES, please expand_________________________________________________________________________________________

**Eight Domains of Good Professional Practice**

**(Medical Council of Ireland 2010)**

**Scholarship**: Doctors must systematically acquire, understand and demonstrate the substantial body of knowledge that is at the forefront of the field of learning in their specialty, as part of a continuum of lifelong learning. They must also search for the best information and evidence to guide their professional practice.

**Professional conduct and ethics**: Doctors must demonstrate a commitment to fulfilling professional responsibilities by adhering to the   standards   specified   in   the   Medical   Council’s   “Guide   to Professional Conduct and Ethics for Registered Medical  Practitioners”.

**Examples of Incidents**

Failure to maintain patient records.

Prescribing incorrect medication to patient.

Lack of communication between staff leading to lack of required care for patient.

Failure to adhere to standard protocols for sterile procedures.

Failure to detect sepsis.

Failure to notice patient deterioration.

Failure to provide information to a patient or their family.

**Clinical Skills**: The maintenance of Professional Competence in the clinical skills domain is clearly specialty-specific and standards should be set by the relevant Post-Graduate Training Body according to international benchmarks.

**Patient safety and quality of patient care**: should be at the core of the health service delivery that a doctor provides. A doctor needs to be accountable to their professional body, to the organisation in which they work, to the Medical Council and to their patients thereby ensuring the patients whom they serve receive the best possible care.

**Management (including Self Management)**: A doctor must understand how working in the health care system, delivering patient care and other professional and personal activities, affect other healthcare professionals, the healthcare system and wider society as a whole.

**Collaboration and Teamwork**: Doctors must co-operate with colleagues and work effectively with healthcare professionals from other disciplines and teams. He/she should ensure that there are clear lines of communication and systems of accountability in place among team members to protect patients.

**Communication and Interpersonal Skills**: Doctors must demonstrate effective interpersonal communication skills. This enables the exchange of information, and allows for effective collaboration with patients, their families and also with clinical and non-clinical colleagues and the broader public.

**Relating to Patients**: Good medical practice is based on a relationship of trust between doctors and society and involves a partnership between patient and doctor that is based on mutual respect, confidentiality, honesty, responsibility and accountability.
