# Supplementary material for: The co-design, implementation and evaluation of a serious board game ‘PlayDecide patient safety’ to educate junior doctors about patient safety and the importance of reporting safety concerns
Source: BMC Med Educ. 2019 Jun 25;19:232. doi: 10.1186/s12909-019-1655-2 (PMC6593521; doi:10.1186/s12909-019-1655-2)
Supplement: Supplementary file 4 — Appendix 4. Voting on Position Statements. Table 1. Hospital A Results: Percentages of how junior doctors voted for each position statement. Table 2. Hospital B Results: Percentages of how junior doctors voted for each position statement. (DOCX 14 kb) [file 12909_2019_1655_MOESM4_ESM.docx]

Appendix 4 Voting on Position Statements

Table 1: Hospital A Results: Percentages of how junior doctors voted for each position statement

|  | **Position 1** | **Position 2** | **Position 3** | **Position 4** |
| --- | --- | --- | --- | --- |
| Support | **75.44%** | 14.81% | 0% | 0% |
| Somewhat Support | 14.04% | 29.63% | 3.45% | 0% |
| Slightly Support | 8.77% | **37.04%** | 6.90% | 0% |
| Slightly Not Acceptable | 1.75% | 11.11% | 18.97% | 5.08% |
| Somewhat Not Acceptable | 0% | 5.56% | 34.48% | 25.42% |
| Not Acceptable | 0% | 1.85% | **36.21%** | **67.80%** |
| Abstain | 0% | 0% | 0% | 1.69% |

*Note: Missing data were excluded from the analysis.*

Table 2: Hospital B Results: Percentages of how junior doctors voted for each position statement

|  | **Position 1** | **Position 2** | **Position 3** | **Position 4** |
| --- | --- | --- | --- | --- |
| Support | **47.6%** | **33.3%** | 0% | 0% |
| Somewhat Support | 40.5% | 12.8% | 5.1% | 0% |
| Slightly Support | 9.5% | 23.1% | 12.8% | 2.6% |
| Slightly Not Acceptable | 2.4% | 15.4% | 15.4% | 12.8% |
| Somewhat Not Acceptable | 0% | 15.4% | **33.3%** | 17.9% |
| Not Acceptable | 0% | 1.85% | 30.8% | **64.1%** |
| Abstain | 0% | 0% | 2.6% | 2.6% |

*Note: Missing data were excluded from the analysis.*
