# Supplementary material for: The co-design, implementation and evaluation of a serious board game ‘PlayDecide patient safety’ to educate junior doctors about patient safety and the importance of reporting safety concerns
Source: BMC Med Educ. 2019 Jun 25;19:232. doi: 10.1186/s12909-019-1655-2 (PMC6593521; doi:10.1186/s12909-019-1655-2)
Supplement: Supplementary file 5 — Appendix 5. Table 1. Item Statistics – Leader Inclusiveness. Table 2. Item Statistics – Psychological safety scale. (DOCX 14 kb) [file 12909_2019_1655_MOESM5_ESM.docx]

| **Appendix 5 Table 1. Item Statistics – Leader Inclusiveness** | | | | |
| --- | --- | --- | --- | --- |
|  | | Hospital A (n=74) mean (standard deviation) | Hospital B (n=71) mean (standard deviation) |  |
|  | Senior doctors encourage other members of the team to take initiative | 5.38 (1.67) | 5.52 (0.98) |  |
|  | Senior doctors ask for the input of team members that belong to other professional groups | 5.93 (1.02) | 5.96 (0.99) |  |
|  | Senior doctors do not value the opinion of others equally | 4.87 (1.6) | 4.13 (1.76) |  |

| **Appendix 5 Table 2. Item Statistics – Psychological safety scale** | | | | |
| --- | --- | --- | --- | --- |
|  | | Hospital A (n=71) mean (sd) | Hospital B (n=69) mean (sd) |  |
|  | Members of this team are able to bring up problems and tough issues | 5.63 (0.97) | 5.49 (1.18) |  |
|  | People in this team are comfortable checking with each other if they have questions about the right way to do something | 5.68 (1.17) | 5.81 (1.09) |  |
|  | If you make a mistake on this team, it is often held against you | 5.31 (1.14) | 5.13 (1.37) |  |
|  | It is difficult to ask other members of this team for help | 5.71 (1.2) | 5.48 (1.46) |  |
|  | Working with members of this team, my unique skills and talents are valued and utilised | 5.04 (1.19) | 4.42 (1.38) |  |
